# Hierarchical coordinated scheduling algorithm for reactive power and voltage in cross-regional power grids based on multi-agent reinforcement learning

**DOI:** 10.1371/journal.pone.0346570

**Published:** 2026-04-24

**Authors:** Zhida Lin, Ximing Zhang, Zhengguo Ren, Yanning Shao, Yuanfeng Chen

**Affiliations:** China Southern Power Grid Company Limited, Guangzhou, Guangdong, China; Dr Shakuntala Misra National Rehabilitation University, INDIA

## Abstract

To address the challenges of strong dynamic coupling, action space dimension explosion, and voltage imbalance in reactive power and voltage scheduling of cross-regional power grids, this paper proposes a hierarchical coordinated scheduling method based on multi-agent reinforcement learning. The method first constructs a multi-agent reinforcement learning framework driven by probabilistic neural networks to perform distributed representation learning on the joint state vectors, achieving high-precision prediction of reactive power and voltage operating states for each node (prediction error MAE < 0.01 p.u.). Building upon the prediction results, a three-layer “prediction-decision-regulation” coordination mechanism is designed, integrating environmental state perception, action space optimization, and dynamic sensitivity analysis. This effectively addresses real-time decision-making challenges in high-dimensional action spaces, reducing average scheduling decision time by approximately 34.2%. Finally, sensitivity-driven feedback regulation achieves real-time balancing of reactive power and voltage at each node, guiding the power grid to converge stably to an optimal power flow state. Experimental results on the IEEE 33-node system demonstrate that the proposed method increases the voltage qualification rate to 98.7%, reduces system power loss by 30.5%, and decreases the maximum voltage magnitude deviation from 1.679 p.u. to 1.589 p.u., significantly outperforming traditional methods.

## 1 Introduction

### 1.1 Research background and motivation

With the rapid development of new power systems, the scale of interconnection among cross-regional power grids continues to expand, posing more complex operational environments and higher safety requirements for reactive power and voltage control. The integration of a high proportion of renewable energy sources intensifies the randomness and volatility of power grid operation, leading to significant disparities in reactive power support capability among different nodes. Consequently, achieving dynamic and balanced allocation of reactive power resources across multiple regions has become a critical and urgent issue to be addressed.

### 1.2 Overview of related work

Traditional centralized dispatch methods struggle to adapt to the dynamic coupling characteristics of multi-regional power grids, while distributed coordination suffers from insufficient information exchange and action conflicts, leading to inadequate voltage fluctuation suppression capabilities and difficulty in maintaining optimal power flow. Current research primarily seeks breakthroughs through two main approaches: model-driven and data-driven methods.

Several model-driven approaches have been proposed for reactive power and voltage scheduling. Zhang et al. [1] developed a two-layer coordinated optimization control strategy based on model predictive control. Their method coordinates frequency and voltage by regulating the active and reactive power of wind farms at the sending end. It employs rolling time domain estimation and an upper-level controller to calculate optimal power values, which are then fed into local wind farm controllers. However, the sensitivity analysis in the upper control layer operates at fixed time intervals, making it difficult to respond to rapid voltage fluctuations.

Huang et al. [2] proposed an online reactive power voltage scheduling method that combines source-load uncertainty with mechanism-data hybrid-driven models. Using mechanism-data hybrid driving theory, they employ both deterministic and stochastic reactive power optimization strategies as training data. A convolutional neural network-gated recurrent unit model captures the influence of source-load uncertainty on reactive power optimization. Nevertheless, this model is primarily trained for single-region source-load fluctuations and lacks the capability to represent cross-regional dynamic coupling characteristics.

Belhamidi et al. [3] utilized wind turbines with variable frequency drives to regulate reactive power in the power grid. Their approach provides or consumes reactive power as needed, employing compensation devices to improve operation. While effective for single wind farm compensation, this method lacks systematic modeling of dynamic coupling characteristics in multi-area power grids. Consequently, it cannot accurately characterize the interactive effects of reactive power support between nodes, limiting the prediction accuracy of the joint state vector and affecting reactive voltage dispatch effectiveness.

Yoo et al. [4] proposed an optimal reactive power dispatch strategy that minimizes power losses under internal voltage layout constraints. Reactive power is generated by wind turbines equipped with power converter systems and allocated to each turbine based on grid reactive power commands. Multiple objective functions are introduced to achieve overall scheduling optimization while considering grid voltage fluctuations. However, this method adopts a centralized optimization architecture, which faces action space explosion when dealing with large-scale grids. It also lacks a distributed decision-making mechanism to handle inter-regional control conflicts, resulting in poor scheduling performance.

In the data-driven approach, reinforcement learning, particularly Multi-Agent Reinforcement Learning (MARL), offers a new paradigm for grid coordination control due to its capabilities in distributed decision-making and online learning. Recent studies have focused on intelligent coordination and optimization of MARL in power systems. For instance, addressing collaboration and communication constraints among multiple agents, Yue et al. [5] proposed an event-triggered distributed coordination strategy, effectively reducing system communication burden and enhancing real-time control. Concurrently, to improve the perception and generalization ability of models regarding grid topology, Jiang et al. [6] explored a framework integrating Graph Neural Networks (GNN) with reinforcement learning to better handle coupling relationships and delay effects between nodes. These advancements have deepened the application of MARL in power systems. However, existing methods still face dual challenges when applied to cross-regional grids: first, the strong electrical coupling and complex dynamic interactions between nodes make high-precision modeling of joint states difficult; second, the high-dimensional discrete action space formed by numerous control devices leads to inefficient traditional exploration strategies and significant decision delays. Consequently, centralized optimization methods are limited by the “curse of dimensionality” and the assumption of global information, while traditional distributed rule-based control lacks adaptive optimization capabilities. Existing MARL methods have yet to effectively integrate accurate state predictors with efficient action decision-making mechanisms, making it challenging to achieve real-time, robust coordinated scheduling under the dual constraints of state coupling and action dimensionality. This limitation precisely represents the key entry point and innovative direction for research.

### 1.3 Major contributions of this study

This paper proposes a hierarchical coordinated scheduling algorithm for reactive power and voltage in cross-regional power grids, driven by multi-agent reinforcement learning. The main contributions are as follows:

(1) A PNN-based joint state prediction framework is proposed. A Probabilistic Neural Network (PNN) is employed as a joint action predictor to conduct distributed representation learning of the dynamic coupling relationships in multi-regional power grids. This effectively addresses the challenge of accurately modeling the states of strongly coupled systems.
(2) A “prediction-decision-regulation” three-layer coordination mechanism is designed. Building upon the state predictions, this mechanism integrates environmental state awareness, action space dimensionality reduction, and dynamic sensitivity feedback to construct a hierarchical closed-loop control structure. This successfully overcomes the difficulties associated with exploring high-dimensional action spaces and making real-time decisions.
(3) A distributed reward and update mechanism tailored for cross-regional grids is constructed. The state space, action space, and a composite reward function are explicitly defined. A policy learning method based on the multi-agent Actor-Critic framework is provided, ensuring the convergence of the algorithm.
(4) The effectiveness of the method is validated through comprehensive experiments. Extensive comparative experiments and statistical analyses were conducted on a standard test system. A complete chain of evidence, including mean values, standard deviations, and convergence curves, is provided to substantiate the performance claims.

### 1.4 Organization of the paper

This paper presents a grid state prediction framework based on multi-agent reinforcement learning and PNN. It elaborates on the three-layer mechanism for hierarchical coordinated dispatch of reactive power and voltage in cross-regional grids. The experimental setup, results, and robustness analysis are also introduced. Finally, the content of this paper is summarized, and potential directions for future work are discussed.

## 2 Grid reactive voltage operating state prediction based on multi-agent reinforcement learning

### 2.1 Key element definitions and multi-agent framework

Addressing the key challenge of accurately modeling the joint state vector due to the strong dynamic coupling between nodes in cross-regional reactive power scheduling, a multi-agent reinforcement learning framework based on probabilistic neural networks is proposed. First, the core elements of the framework are explicitly defined:

(1) State Vector: The observed state of agent i is denoted as $s^{i}=[V_{i}\;,cos\phi_{i}\;,P_{i}\;,Q_{i\;}{]}^{T}$, and the joint state is represented by $s\in R^{N\times 4}$, where N is the number of agents (or key nodes).
(2) Action Vector: The discrete action set for agent i is defined as $A_{i}=\left\{a_{\text{1}}\;:\text{Investment capacitor},a_{\text{2}}\;:\text{Cut capacitor},a_{\text{3}}\;:\text{Lift tap connector},a_{\text{4}}\;:\text{Drop connector},a_{\text{5}}\;:\text{maintain}\right\}$.
(3) Reward Function: A composite reward function is designed to guide global optimization:

$r_{t\;}=-\;\alpha \sum_{i=1}^{N}(V_{i}\;-V_{\text{ref}}\;{)}^{\text{2}}+\beta P_{\text{loss,t}}+\gamma \sum_{j\in V_{\text{vio}}}\;I_{\text{penalty}}\;$, where $\begin{array}{c} \;\\ \alpha \text{,}\beta \text{,}\gamma \end{array}$ are weighting coefficients, $\;V_{\text{vio}}$ is the set of nodes with voltage violations, and $I_{\text{penalty}}$ is the penalty term for violations. This function simultaneously optimizes voltage quality, reduces network losses, and ensures safety.

A multi-agent system (MAS) consists of multiple independent autonomous agents [7] operating in the same working environment, capable of perceiving environmental information and executing their respective behaviors. Applying MAS to cross-regional power grid reactive power voltage coordinated dispatch enables the acquisition of data on the operational status and voltage of different devices, thereby facilitating coordinated dispatch. The generalized Markov process of MAS can be regarded as a random strategy, defined as follows: a random strategy can be represented by the tuple ⟨N,S,A,T,R⟩, where, N denotes the number of agents in the system, S denotes the set of environmental states, $A^{i}$ denotes the set of actions available to agent i=1,2,⋯,N, and the joint action set is denoted as: $A=A^{\text{1}}\times \cdots \times A^{N}$. T:S×A×S→[0,1] denotes the state transition probability function, and R:S×A×S→ℝ indicates reward function [8]. In MAS, state transitions are the result of the joint actions of all agents in the system. The reinforcement signal function also depends on joint actions, and the mapping strategy from state to action is extended to a joint strategy.

### 2.2 Joint action prediction based on Probabilistic Neural Network (PNN)

**Fig 1:**
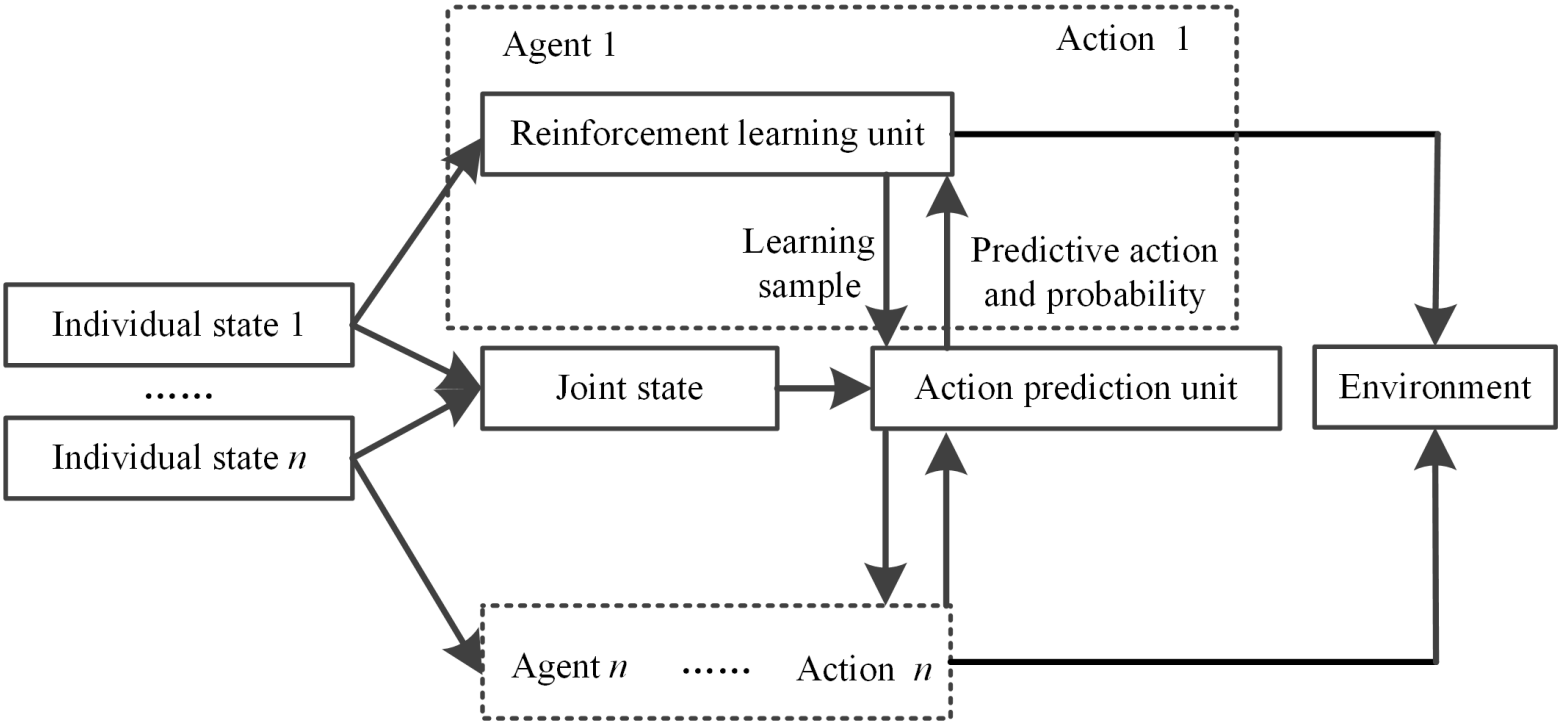
Structure of the multi-agent reinforcement learning system integrated with a PNN predictor.

In partially observable multi-agent environments, agents cannot directly access the real-time actions of other agents, leading to decision-making based on incomplete information. To address this issue, a probabilistic neural network is introduced as a joint action predictor. The PNN is not directly used for policy approximation; instead, it leverages its strong pattern classification and probability density estimation capabilities. Based on the current joint state $s_{t}$, it predicts the probability distribution $\hat{P}\left(\overset{j}{\underset{t}{a}}|s_{t}\right)$ of possible actions that other agents j≠i might take. This prediction information serves as additional input to agent i ’s own policy, thereby significantly enhancing the coordination of multi-agent decision-making while reducing communication requirements. The system composition is illustrated in Fig 1.

Q learning is one of the most important reinforcement learning algorithms. Based on the above definitions, the two-agent learning method based on general and policy-based reinforcement learning Q is extended to multi-agent learning Q. The function Q depends on the actions executed by all agents, so the function update rule for agents at time t can be expressed as:


$$\overset{i}{\underset{t}{Q}}\left(\overset{i}{\underset{t}{S}},\vec{A^{i}}\right)=\left(1-A^{i}\right)\overset{i}{\underset{t+1}{Q}}\left(\overset{i}{\underset{t}{S}},\vec{A^{i}}\right)+A^{i}\left[\overset{i}{\underset{t}{r}}\left(\overset{i}{\underset{t}{S}},\vec{A^{i}}\right)+\beta \pi^{\text{1}}\left({\vec{S}}_{t+1}\right)\cdots \pi^{n}\left({\vec{S}}_{t+1}\right)\overset{i}{\underset{t-1}{Q}}\left({\vec{S}}_{t+1}\right)\right]$$ (1)

$$\pi^{\text{1}}\left({\vec{S}}_{t+1}\right)\cdots \pi^{n}\left({\vec{S}}_{t+1}\right)\overset{i}{\underset{t}{Q}}\left({\vec{S}}_{t+1}\right)=\sum_{A^{\text{1}}}\cdots \sum_{A^{n}}\overset{\text{1}}{\underset{t}{P}}\left({\vec{S}}_{t+1},A^{\text{1}}\right)\cdots \overset{n}{\underset{t}{P}}\left({\vec{S}}_{t+1},A^{n}\right)\overset{i}{\underset{t-1}{Q}}\left({\vec{S}}_{t-1},A^{\text{1}},\cdots ,A^{n}\right)$$ (2)

Where, ${\vec{S}}_{t+1}$ and ${\vec{S}}_{t-1}$ represent the joint state of multiple agents at the next and previous time steps, respectively. The state transition of individual agents is determined by the function $S_{t+1}=\overset{i}{\underset{t}{f}}\left(S_{t},\vec{A}\right)$; $\pi^{i}$ denotes the probability distribution strategy for the action set of agent i[9]; $\overset{i}{\underset{t}{P}}\left({\vec{S}}_{t+1},A^{i}\right)$ represents the probability of agent i selecting $A^{i}$ under the joint state ${\vec{S}}_{t+1}$; β denotes the state variables of agent i; $\overset{i}{\underset{t}{r}}$ denotes the reinforcement signal.

Adopting a centralized training and distributed execution paradigm based on the Actor Critic framework. In the multi-agent Q learning rules described in equations (1) and (2), the Actor Critic framework plays an important role. During centralized training, by collecting and analyzing global information such as joint states, joint rewards, etc., the Critic part (i.e., Q function) is used to evaluate the value of the agent’s actions, guiding the Actor part (i.e., the agent’s strategy) to update and optimize the overall strategy.

From the above analysis, it can be seen that the Q learning algorithm redefines the function of the state-action pair $\left(S^{t},A^{t}\right)$ as the Q function of the intelligent agent’s state and combined action $\left(\overset{i}{\underset{t}{S}},{\vec{A}}_{t}\right)$. Therefore, the key issue in multi-agent reinforcement learning is how to determine the joint state and joint action of multiple agents. Since multiple agents select actions simultaneously, each agent cannot know the actions of other agents, making it difficult to precisely determine the joint action. However, in most learning problems, the action selection strategy follows a certain probability distribution of other agents’ behaviors [10]. Therefore, the multi-agent reinforcement learning system constructed consists of an action prediction unit and a reinforcement learning unit.

In the multi-agent reinforcement learning system, the action prediction unit is implemented using probabilistic neural network methods and provides the reinforcement learning unit with the actions selected by other agents and their predicted probabilities, thereby completing the multi-agent reinforcement learning algorithm. The reinforcement learning unit returns the accumulated learning examples to the action prediction unit to update the prediction model.

**Fig 2:**
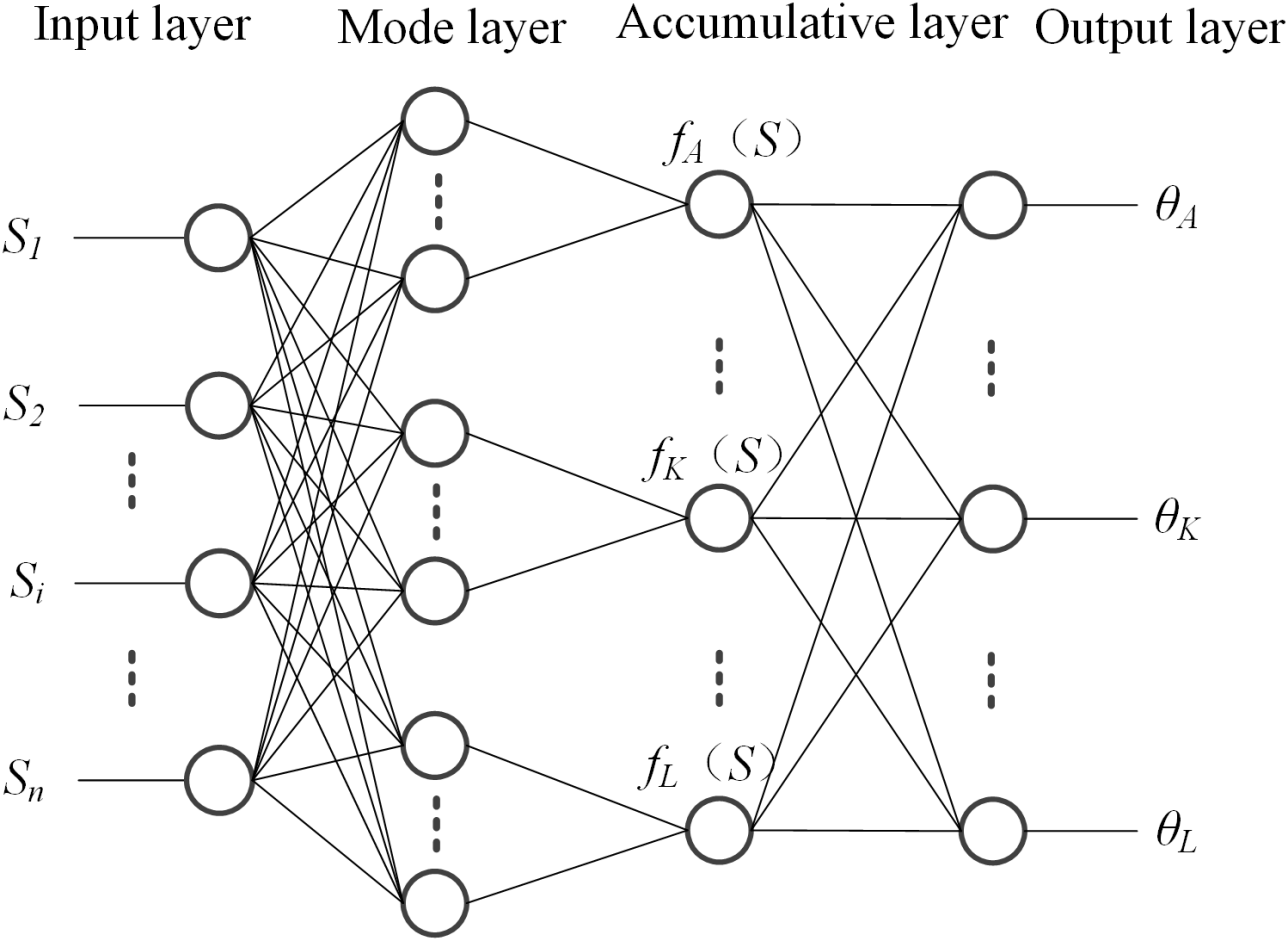
Probabilistic neural network structure diagram.

After analyzing the multi-agent reinforcement learning unit, the action prediction unit is analyzed to obtain more accurate and specific operational status of electrical equipment in different grid areas. Multi-agent action prediction is implemented using a probabilistic neural network. A probabilistic neural network is a neural network model used for classification, which applies Bayesian decision analysis theory estimated by the Parzen window [11]. Its network structure is shown in Fig 2.

The implementation process of agent action prediction based on PNN is as follows: first, the joint state of multi-agents $\vec{S}$ is used as the input vector of the network [12], and the candidate actions of the agents are used as the decision categories $\theta_{A},\cdots ,\theta_{K},\cdots ,\theta_{L}$ of the network output; then, the probabilities of selecting actions under the joint state are obtained through network inference. PNN is a four-layer feedforward network structure, consisting of an input layer, a pattern layer, an accumulation layer, and an output layer. The input layer directly transmits the input vector $\vec{S}$ to the nodes of the pattern layer; the pattern layer performs a weighted sum of the input $\vec{S}$ and the given class weight vector $\omega_{j}$ [13], then applies a nonlinear operation to the result and passes it to the accumulation layer; The accumulation layer accumulates the probabilities of $\vec{S}$ belonging to the same category and passes the result to the decision layer; the neurons in the decision layer are competitive neurons [14], which receive the probability density functions of various categories from the accumulation layer.

The aforementioned PNN is fully equivalent to the Bayesian pattern classification method using a multivariate probability density function with a Gaussian kernel [15]. The probability density function of the input vector belonging to a class is calculated as follows [16]:


$$f_{K}\left(\vec{S}\right)=\frac{\text{1}}{{\left(2\pi \right)}^{M/2}\delta^{M}}\cdot \frac{\text{1}}{n_{K}}\sum_{j=1}^{n_{K}}exp\left[-\frac{{\left(\vec{S}-{\vec{S}}_{K_{j}}\right)}^{T}\left(\vec{S}-{\vec{S}}_{K_{j}}\right)}{2\delta^{\text{2}}}\right]$$ (3)

Where, M denotes the number of components in the input pattern vector, ${\vec{S}}_{K_{j}}$ denotes the j th training sample vector belonging to the class K, δ denotes the smoothing coefficient used to adjust the density function, T denotes the time vector, and $n_{K}$ denotes the number of training sample vectors in the class K.

According to equation (3), the probability $f_{K}\left(\vec{S}\right)$ that $\vec{S}$ belongs to the K class can be calculated. The PNN decision uses the Bayesian decision criterion [17] to determine the state of the $\theta \in \theta_{Q}$ class, which can be expressed as:


$$d\left(\vec{S}\right)\in \theta_{Q},if\text{\textbackslash{}hspace\{0.5em}\;\;\}h_{Q}l_{Q}f_{Q}\left(S\right)≻h_{K}l_{K}f_{K}\left(S\right)$$ (4)

Where, $d\left(\vec{S}\right)$ denotes the Bayesian decision for the test vector, $h_{Q}$ and $h_{K}$ denote the prior probabilities of categories $\theta \in \theta_{Q}$ and $\theta \in \theta_{K}$, respectively, $l_{Q}$ and $l_{K}$ denote the losses incurred when the categories $\theta \in \theta_{Q}$ and $\theta \in \theta_{K}$ are misclassified [18], and $f_{Q}$ and $f_{K}$ denote the probability density functions of categories $\theta \in \theta_{Q}$ and $\theta \in \theta_{K}$, respectively.

During the reinforcement learning process, each learning example is continuously added to the corresponding group in the pattern layer, while the number of samples belonging to each category is updated. Under the joint state $\vec{S}$, the conditional probability of the agent selecting action $A_{K}$ is:


$$P\left(A_{K}|\vec{S}\right)\propto h_{K}l_{K}f_{K}\left(\vec{S}\right)$$ (5)

In multi-agent reinforcement learning, the joint state $\vec{S}$ serves as the input pattern vector for the PNN, with the number of input layer nodes determined by the number of components in $\vec{S}$. The agent’s action space serves as the decision category, and the number of candidate actions determines the number of nodes in the accumulation layer. Therefore, the agent action prediction problem can be viewed as the process of classifying the input joint state vector using a PNN [19]. The action prediction unit and reinforcement learning unit operate simultaneously to achieve overall action prediction and action selection. The PNN approach is applied to the hierarchical coordinated scheduling of reactive power and voltage in cross-regional power grids. By predicting the reactive power and voltage states of other grid layers, their predicted operational conditions are obtained. Based on the prediction results, the voltages of other layers are adjusted and controlled to achieve optimal coordinated scheduling. Assume that there are m nodes in the power grid, each with p state variables (such as voltage magnitude, phase, reactive power, etc.) describing its operational state. The joint state vector $\overset{―}{S}$ can be represented as $\overset{―}{S}=\left[S_{\text{1}},S_{\text{2}},\cdots ,S_{m}\right]$, where $S_{i}=\left[s_{i1},s_{i2},\cdots ,s_{ip}\right]$(i=1,2,⋯,n). Each agent has q candidate actions for each node (e.g., $a_{\text{1}}$: increase reactive power compensation; $a_{\text{2}}$: decrease reactive power compensation;  ...; $a_{q}$: maintain the current state). The probability $P(a_{k}|\overset{―}{S})$ of selecting action $a_{k}(k=1,2,\cdots ,q)$ under the joint state $\overset{―}{S}$ is calculated using PNN. Actions are selected based on the principle of maximum probability or other strategies to achieve prediction and regulation of the reactive voltage operating states of all nodes in the power grid.

In summary, the multi-agent reinforcement learning framework based on probabilistic neural networks achieves effective control of the reactive voltage prediction operating states of all grid nodes through training and action prediction of the joint state vector of multiple agents, providing strong support for reactive voltage coordination scheduling in cross-regional power grids.

## 3 Cross-regional power grid reactive voltage hierarchical collaborative dispatch

**Fig 3:**
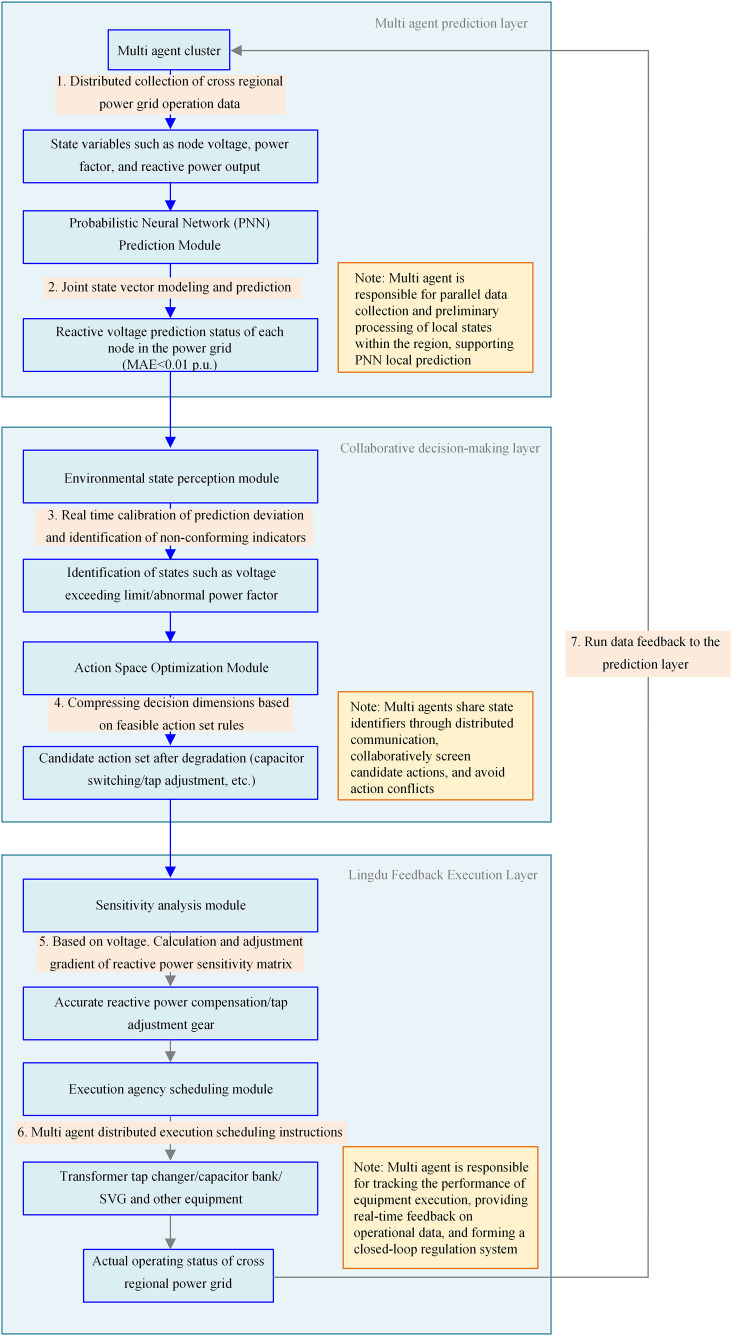
Schematic Diagram of Hierarchical Coordinated Scheduling for Reactive Power and Voltage in Cross-Regional Power Grids Driven by Multi-Agent Reinforcement Learning.

Although the prediction model can accurately capture the operational status of the power grid, its direct application still faces issues such as decision-making efficiency degradation due to the explosion of action space dimensions, and the lack of adaptive regulatory capabilities for the dynamic coupling characteristics of the power grid. To address this, it is necessary to real-time calibrate prediction errors through an environmental state perception module, compress decision dimensions using action space optimization techniques, and establish a dynamic feedback mechanism based on sensitivity analysis, forming a closed-loop control system of “prediction-decision-regulation” (a schematic diagram of which is shown in Fig 3). This method inherits the state recognition advantages of prediction models while addressing the real-time decision-making challenges of high-dimensional action spaces through a coordinated scheduling mechanism. Ultimately, it achieves precise coordinated control of reactive power and voltage across regional power grids, enabling the system to stabilize and converge to its optimal operating state.

### 3.1 Reactive power voltage coordinated scheduling based on environmental state and actionable actions

(1) Set of environmental states

For the reactive power voltage layered coordinated dispatch problem, the environmental state, i.e., the operational state of the power grid, can be represented by the electrical quantities to be evaluated in the regional power grid. Here, the power factor of the injected power at the nodes [20] and the voltage amplitude at the nodes are selected as the state variables. For computational convenience, all quantities are normalized as follows:


$$\eta_{m}=P(a_{k}|\overset{―}{S})\left(\frac{x_{m}-x_{m,min}}{x_{m,max}-x_{m,min}}\right)$$ (6)

Where, $x_{m}$ denotes the m th electrical state indicator to be evaluated, $x_{m,max}$ and $x_{m,min}$ denote the upper and lower limits of the evaluation indicator in the normal operating state, respectively; $\eta_{m}$ denotes the normalized result. When $\eta_{m}≻1$, the indicator is closer to the upper limit; when $\eta_{m}≺0$, the indicator is closer to the lower limit; when $0\leq \eta_{m}\leq 1$, the indicator is within the acceptable range; and when $\eta_{m}=0.5$, the indicator reaches the optimal state.

Further classification of the indicator states $\eta_{m}$ is performed. The finer the state classification, the more accurately the power grid operating condition is described; however, overly fine state classification leads to an excessive number of elements in the environmental state set, resulting in a longer learning cycle [21], which is detrimental to online control analysis. After comprehensive consideration, each electrical performance indicator is classified into 7 states, as shown in Table 1.

**Table 1:** Indicator state classification.

$\eta_{m}$Scope	Indicator Status $y_{m}$
(- ∞ , 0)	1
(0, 0.2)	2
(0.2, 0.4)	3
(0.4, 0.6)	4
(0.6, 0.8)	5
(0.8, 1.0)	6
(1.0, +∞)	7

In Table 1, indicator states 1 and 7 represent the lower and upper limits of the indicator, respectively. In practical applications, to ensure system safety, limit values can be set based on the principle of maintaining a small safety margin relative to the on-site safety threshold. The set of indicator states that meet the criteria is denoted as $y_{m}\in \left\{2,3,4,5,6\right\}$, where state 4 represents the optimal state, and the remaining states deteriorate in quality as the distance from state 4 increases. For regional power grids containing m electrical quantities under evaluation, the environmental state set S contains a total of ${\text{7}}^{m}$states, each of which can be represented as $s=\left\{y_{\text{1}},y_{\text{2}},\cdots ,y_{m}\right\}$.

(2) Action Set

By analyzing the action set for reactive voltage in cross-regional power grids, we can better predict their operational status, address the issue of action space explosion, and further improve the rationality of reactive voltage dispatch between different regions. The reactive voltage coordination dispatch device includes transformer tap changers and capacitor banks. The action set for hierarchical reactive voltage coordination dispatch in cross-regional power grids is defined as: when the power grid is in a certain state s, the set of action strategies that can transition s to a better state s'. According to the online control regulations for reactive power and voltage in power grids, reactive power and voltage control devices are only regulated and dispatched when an environmental state contains non-compliant indicators. Each grid environmental state containing non-compliant evaluation indicators has a corresponding set of actionable measures, and the sets of actionable measures for different environmental states are generally different.

According to on-site operational requirements, the non-compliant electrical indicators requiring reactive voltage optimization regulation and dispatch are categorized into four types: voltage exceeding the upper limit [22], voltage below the lower limit, transformer high-voltage winding power factor exceeding the upper limit, and transformer high-voltage winding power factor below the lower limit. According to on-site operational regulations, at any given time, no more than one device is allowed to act simultaneously within the network connected to each 220 kV feeder. Therefore, for each grid state requiring regulation and dispatch, the action set is determined based on the following four principles:

(1) Voltage exceeds the upper limit: The set of actionable actions is to disconnect capacitors and lower the tap settings of transformers at the station and the previous-level substation where the voltage indicator value $\eta_{m}$ is greater than 0.3 (the indicator is within the normal range and there is a margin before reaching the voltage lower limit);
(2) Voltage exceeds lower limit: The set of actionable operations is to close capacitors and increase transformer tap settings at the current substation and the upstream substation where the voltage indicator value $\eta_{m}$ is less than 0.7 (the indicator is within the normal range and there is still a margin to the voltage upper limit);
(3) Power factor of the high-voltage winding of the transformer exceeds the upper limit: The action set can be executed to disconnect capacitors on the busbar where the voltage indicator value is greater than 0.3 at this station and the lower-level substation;
(4) When the power factor of the high-voltage winding of the transformer exceeds the lower limit: the action set includes energizing capacitors on the busbar where the voltage indicator value is less than 0.7 at this station and its subordinate substations.

The determination principles for the above action sets fully consider the actual voltage and power factor conditions of the substation and the transformer. Capacitors and transformers with a sufficient (30%) adjustable margin relative to the limit values are selected for regulation. Among these, energizing capacitors increases the voltage amplitude and power factor of the target voltage, while de-energizing capacitors has the opposite effect; Raising the tap settings of transformers can increase the voltage amplitude of the monitored voltage, while lowering the tap settings has the opposite effect. The purpose of reinforcement learning is to establish the optimal association between states within the state set and actions within the action set for a distributed power grid through continuous interaction with the environment.

### 3.2 Reactive power and voltage coordinated dispatch based on sensitivity analysis

Static Var Generators (SVGs) have the characteristics of rapid and smooth regulation of reactive power output, making this compensation method highly suitable for applications with frequent voltage fluctuations. When the system voltage exceeds the boundary, to determine the relationship between the voltage increment at each node in the grid ΔU and the reactive power compensation capacity ΔO, the voltage-reactive power sensitivity matrix of the system can be derived using the Newton-Raphson method’s power flow calculation equations. The power flow equations for a cross-regional grid are transformed into the following form:


$$\left[\begin{array}{c} @l@\Delta W_{\text{1}}\\ \Delta O_{\text{1}}\\ \Delta W_{\text{2}}\\ \Delta O_{\text{2}}\\ \cdots \\ \cdots \\ \Delta W_{b-1}\\ \Delta O_{b-1} \end{array}\right]=J\eta_{m}\left[\begin{array}{c} @l@\Delta e_{\text{1}}\\ \Delta v_{\text{1}}\\ \Delta e_{\text{2}}\\ \Delta v_{\text{2}}\\ \cdots \\ \cdots \\ \Delta e_{b-1}\\ \Delta v_{b-1} \end{array}\right]$$ (7)

$$\begin{array}{c} J=\left[\begin{array}{cccccc} \frac{\partial \Delta W_{\text{1}}}{\partial e_{\text{1}}} & \frac{\partial \Delta W_{\text{1}}}{\partial v_{\text{1}}} & \frac{\partial \Delta W_{\text{1}}}{\partial e_{\text{2}}} & \frac{\partial \Delta W_{\text{1}}}{\partial v_{\text{2}}}\cdots & \cdots \frac{\partial \Delta W_{\text{1}}}{\partial e_{b-1}} & \frac{\partial \Delta W_{\text{1}}}{\partial v_{b-1}}\\ \frac{\partial \Delta O_{\text{1}}}{\partial e_{\text{1}}} & \frac{\partial \Delta O_{\text{1}}}{\partial v_{\text{1}}} & \frac{\partial \Delta O_{\text{1}}}{\partial e_{\text{2}}} & \frac{\partial \Delta O_{\text{1}}}{\partial v_{\text{2}}}\cdots & \cdots \frac{\partial \Delta O_{\text{1}}}{\partial e_{b-1}} & \frac{\partial \Delta O_{\text{1}}}{\partial v_{b-1}}\\ \frac{\partial \Delta W_{\text{2}}}{\partial e_{\text{1}}} & \frac{\partial \Delta W_{\text{2}}}{\partial v_{\text{1}}} & \frac{\partial \Delta W_{\text{2}}}{\partial e_{\text{2}}} & \frac{\partial \Delta W_{\text{2}}}{\partial v_{\text{2}}}\cdots & \cdots \frac{\partial \Delta W_{\text{2}}}{\partial e_{b-1}} & \frac{\partial \Delta W_{\text{2}}}{\partial v_{b-1}}\\ \frac{\partial \Delta O_{\text{2}}}{\partial e_{\text{1}}} & \frac{\partial \Delta O_{\text{2}}}{\partial v_{\text{1}}} & \frac{\partial \Delta O_{\text{2}}}{\partial e_{\text{2}}} & \frac{\partial \Delta O_{\text{2}}}{\partial v_{\text{2}}}\cdots & \cdots \frac{\partial \Delta O_{\text{2}}}{\partial e_{b-1}} & \frac{\partial \Delta O_{\text{2}}}{\partial v_{b-1}}\\ \cdots & \cdots & \cdots & \cdots & \cdots & \cdots \\ \cdots & \cdots & \cdots & \cdots & \cdots & \cdots \\ \frac{\partial \Delta W_{b-1}}{\partial e_{\text{1}}} & \frac{\partial \Delta W_{b-1}}{\partial v_{\text{1}}} & \frac{\partial \Delta W_{b-1}}{\partial e_{\text{2}}} & \frac{\partial \Delta W_{b-1}}{\partial v_{\text{2}}}\cdots & \cdots \frac{\partial \Delta W_{b-1}}{\partial e_{b-1}} & \frac{\partial \Delta W_{b-1}}{\partial v_{b-1}}\\ \frac{\partial \Delta O_{b-1}}{\partial e_{\text{1}}} & \frac{\partial \Delta O_{b-1}}{\partial v_{\text{1}}} & \frac{\partial \Delta O_{b-1}}{\partial e_{\text{2}}} & \frac{\partial \Delta O_{b-1}}{\partial v_{\text{2}}}\cdots & \cdots \frac{\partial \Delta O_{b-1}}{\partial e_{b-1}} & \frac{\partial \Delta O_{b-1}}{\partial v_{b-1}} \end{array}\right] \end{array}$$ (8)

In the equation, J denotes the Jacobian matrix, b denotes the total number of nodes, W and O denote grid nodes, respectively, e and v denote reactive voltage nodes, respectively.

For the voltage $U_{b}$ of node b in a cross-regional power grid, it can be expressed in the following form:


$$U_{b}=\sqrt{\overset{\text{2}}{\underset{b}{e}}+\overset{\text{2}}{\underset{b}{v}}}$$ (9)

Differentiating the above equation yields:


$$DU_{b}=\frac{e_{b}De_{b}}{\sqrt{\overset{\text{2}}{\underset{b}{e}}+\overset{\text{2}}{\underset{b}{v}}}}+\frac{v_{b}Dv_{b}}{\sqrt{\overset{\text{2}}{\underset{b}{e}}+\overset{\text{2}}{\underset{b}{v}}}}=\frac{e_{b}De_{b}+v_{b}Dv_{b}}{U_{b}}$$ (10)

Where, D represents the differential coefficients. The derivation process of this formula is as follows:

Assuming $f\left(x,y\right)=\sqrt{x^{\text{2}}+y^{\text{2}}}$, according to the derivative rule of composite functions $\frac{\partial f}{\partial x}=\frac{x}{\sqrt{x^{\text{2}}+y^{\text{2}}}}$, $\frac{\partial f}{\partial y}=\frac{y}{\sqrt{x^{\text{2}}+y^{\text{2}}}}$。

For $U_{b}=\sqrt{\overset{\text{2}}{\underset{b}{e}}+\overset{\text{2}}{\underset{b}{v}}}$, let $x=e_{b}$, $y=v_{b}$, then $DU_{b}=\frac{\partial U_{b}}{\partial e_{b}}De_{b}+\frac{\partial U_{b}}{\partial v_{b}}Dv_{b}=\frac{e_{b}}{\sqrt{\overset{\text{2}}{\underset{b}{e}}+\overset{\text{2}}{\underset{b}{v}}}}De_{b}+\frac{v_{b}}{\sqrt{\overset{\text{2}}{\underset{b}{e}}+\overset{\text{2}}{\underset{b}{v}}}}Dv_{b}=\frac{e_{b}De_{b}+v_{b}Dv_{b}}{U_{b}}$. Equations (7), (8), (9), (11), (12), and (13) are derived based on the Newton Raphson method and the basic principles of tidal current calculation.

Expressing the voltages of all nodes in the cross-regional power grid using the above equation and converting them into matrix form yields:


$$\begin{array}{c} \left[\begin{array}{c} @l@\Delta U_{\text{1}}\\ \Delta U_{\text{2}}\\ \cdots \\ \Delta U_{b-1} \end{array}\right]=\left[\begin{array}{c} @l@\frac{e_{\text{1}}}{U_{\text{1}}}\begin{array}{cc} & \frac{v_{\text{1}}}{U_{\text{1}}} \end{array}\\ \begin{array}{cccc} & & & \begin{array}{cc} & \frac{e_{\text{2}}}{U_{\text{2}}}\begin{array}{cc} & \frac{v_{\text{2}}}{U_{\text{2}}} \end{array} \end{array} \end{array}\\ \begin{array}{cccc} & & & \cdots \end{array}\\ \begin{array}{cccc} & & & \begin{array}{cccc} & & & \frac{e_{b-1}}{U_{b-1}}\begin{array}{cc} & \frac{v_{b-1}}{U_{b-1}} \end{array} \end{array} \end{array} \end{array}\right]\left[\begin{array}{c} @l@\Delta e_{\text{1}}\\ \Delta v_{\text{1}}\\ \Delta e_{\text{2}}\\ \Delta v_{\text{2}}\\ \cdots \\ \Delta e_{b-1}\\ \Delta v_{b-1} \end{array}\right] \end{array}$$ (11)

Let the coefficient matrix of the above equation be Z, we have:


$$\left[\begin{array}{c} @l@\Delta U_{\text{1}}\\ \Delta U_{\text{2}}\\ \cdots \\ \Delta U_{b-1} \end{array}\right]=Z\left[\begin{array}{c} @l@\Delta e_{\text{1}}\\ \Delta v_{\text{1}}\\ \Delta e_{\text{2}}\\ \Delta v_{\text{2}}\\ \cdots \\ \Delta e_{b-1}\\ \Delta v_{b-1} \end{array}\right]$$ (12)

Reactive power and voltage coordination is achieved by adjusting static reactive power sources. Therefore, it can be assumed that the active power changes at the nodes are zero during reactive power and voltage scheduling. Combining Equations (7) and (12), the power system converges stably to the optimal power flow operating state, and substituting ΔW=0 yields the final solution for efficient reactive power and voltage scheduling in the inter-regional power grid:


$$\left[\begin{array}{c} @l@\Delta U_{\text{1}}\\ \Delta U_{\text{2}}\\ \cdots \\ \Delta U_{b-1} \end{array}\right]=ZJ^{-1}\left[\begin{array}{c} @l@0\\ \Delta O_{\text{1}}\\ 0\\ \Delta O_{\text{2}}\\ \cdots \\ 0\\ \Delta O_{b-1} \end{array}\right]$$ (13)

The coefficient matrix $ZJ^{-1}$ reflects the sensitivity of the reactive power changes at each node to the reactive power changes at other nodes, i.e., the voltage-reactive power sensitivity matrix. Inter-regional reactive power and voltage coordination scheduling optimizes the reactive power output of capacitor banks and the tap positions of transformers in the network based on the scheduling cycle to achieve the optimal power flow across the entire grid. When the system voltage exceeds the boundary, the reactive voltage scheduling strategy based on environmental status and available actions, and the reactive voltage scheduling strategy based on sensitivity analysis, promptly restore the grid voltage to achieve balance among all nodes.

## 4 Experimental testing

### 4.1 Experimental environment

**Fig 4:**
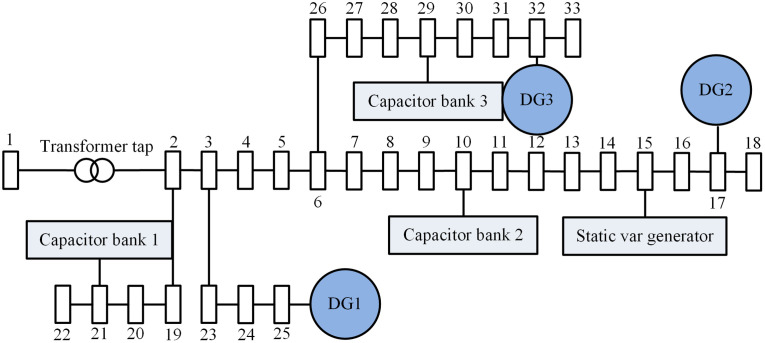
IEEE 33-node power distribution network topology.

To verify whether the proposed method can achieve coordinated scheduling of reactive power and voltage in cross-regional power grids in practical applications, comparative experiments were conducted against the two-layer coordinated optimal control strategy (MPC), the optimal reactive power dispatch strategy (OPF), and the baseline single-agent Deep Q-Network (DQN) algorithm mentioned in the introduction. The experiments were carried out on the MATLAB/Simulink R2023a platform using a 33-node distribution network model. In this model, Node 1 serves as the slack bus with an initial voltage of 1.05 (per unit). A transformer tap changer is installed between Nodes 1 and 2, with a standard turns ratio of 1, 11 tap positions in total, and tap ratio limits of 1.025 and 0.975. Capacitor banks are installed at Nodes 10, 12, and 29, with an adjustment range of 0–0.12 and a step size of 0.03. Distributed generation units are connected at Nodes 17, 25, and 32. A Static Var Generator (SVG) is installed at Node 15. All data in the experiments were analyzed and processed using MATLAB. The dynamic simulation time step was set to 10 ms, the optimal calculation period to 1 minute, the communication delay to 5 ms, and the coordination period to 10 minutes. To evaluate the robustness of the algorithm, 30 independent random experiments were conducted for each method, and the results are reported as “mean ± standard deviation.” The topology of the IEEE 33-node distribution network is shown in Fig 4.

To ensure the reproducibility of the proposed method, the key implementation details are as follows. The probabilistic neural network (PNN) predictor adopts a smoothing coefficient set to 0.1, which was empirically determined through cross-validation to balance prediction accuracy and generalization capability. The multi-agent Actor-Critic framework adopts a centralized training with decentralized execution paradigm. The Actor network for each agent consists of two hidden layers with 128 and 64 neurons, respectively, both using ReLU activation functions. The Critic network comprises three hidden layers with 256, 128, and 64 neurons, also employing ReLU activations. The learning rate for both Actor and Critic networks is set to 10^-^³, with an Adam optimizer used for gradient-based optimization. The discount factor is set to 0.95, and the soft update coefficient for target network synchronization is 0.01. The experience replay buffer size is 10⁵, with a mini-batch size of 64. The exploration-exploitation trade-off is managed using an ε-greedy policy with ε initially set to 1.0 and decaying exponentially to 0.01 over 500 episodes. The random seed for all experiments is fixed at 42 to ensure result reproducibility. All simulations were conducted using MATLAB R2023a with the following toolboxes: Statistics and Machine Learning Toolbox (for PNN implementation), Deep Learning Toolbox (for neural network construction), Optimization Toolbox (for sensitivity analysis), and Simulink (for power system dynamic simulation).

### 4.2 Experimental process and results analysis

**Fig 5:**
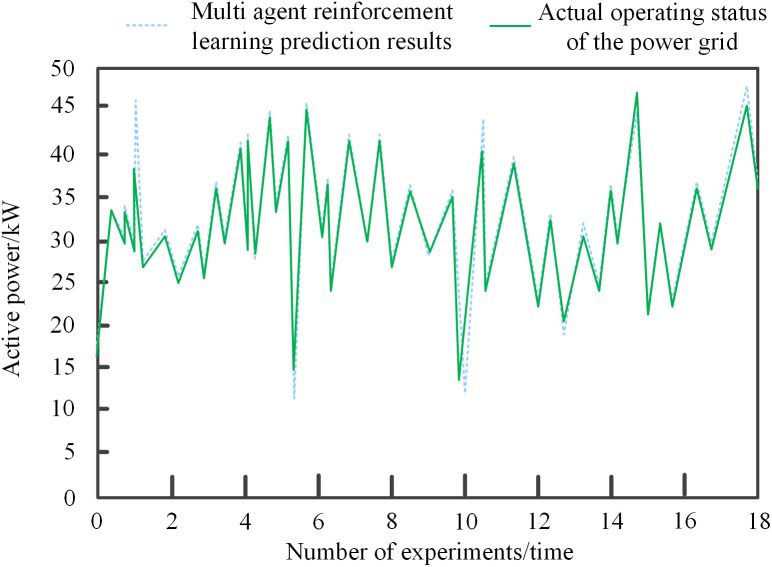
Multi-agent reinforcement learning grid state prediction results.

To validate the effectiveness of the step “based on prediction results, coordinating scheduling from three aspects: environmental state perception, action space optimization, and sensitivity dynamic analysis” in the proposed method, experimental tests were conducted on the grid state prediction performance of multi-agent reinforcement learning to ensure the subsequent completion of reasonable and effective hierarchical coordinated scheduling of reactive power and voltage across regional grids. The results of the multi-agent reinforcement learning grid state prediction are shown in Fig 5.

As shown in Fig 5, the multi-agent reinforcement learning grid state prediction results demonstrate the strong prediction capability of the proposed multi-agent reinforcement learning algorithm. The prediction curve closely aligns with the actual grid operation state curve, with multiple instances of overlap. This indicates that the algorithm can accurately capture the complex dynamic characteristics of the grid operation process, achieving high-precision prediction for key indicators such as voltage fluctuations and power changes. This accurate prediction capability provides a reliable basis for subsequent environmental state perception, enabling action space optimization and sensitivity dynamic analysis to be conducted based on accurate power grid state information. For example, when perceiving a sudden change in load in a certain area of the power grid, the prediction algorithm can quickly predict its impact on the voltage and reactive power distribution of surrounding nodes, thereby providing a clear direction for action space optimization and precise basic data for sensitivity dynamic analysis. Therefore, the precise grid state prediction achieved by the multi-agent reinforcement learning algorithm effectively ensures the validity of the core step of collaborative scheduling based on prediction results, laying a solid foundation for achieving reasonable and effective hierarchical collaborative scheduling of reactive power and voltage across regional power grids.

**Fig 6:**
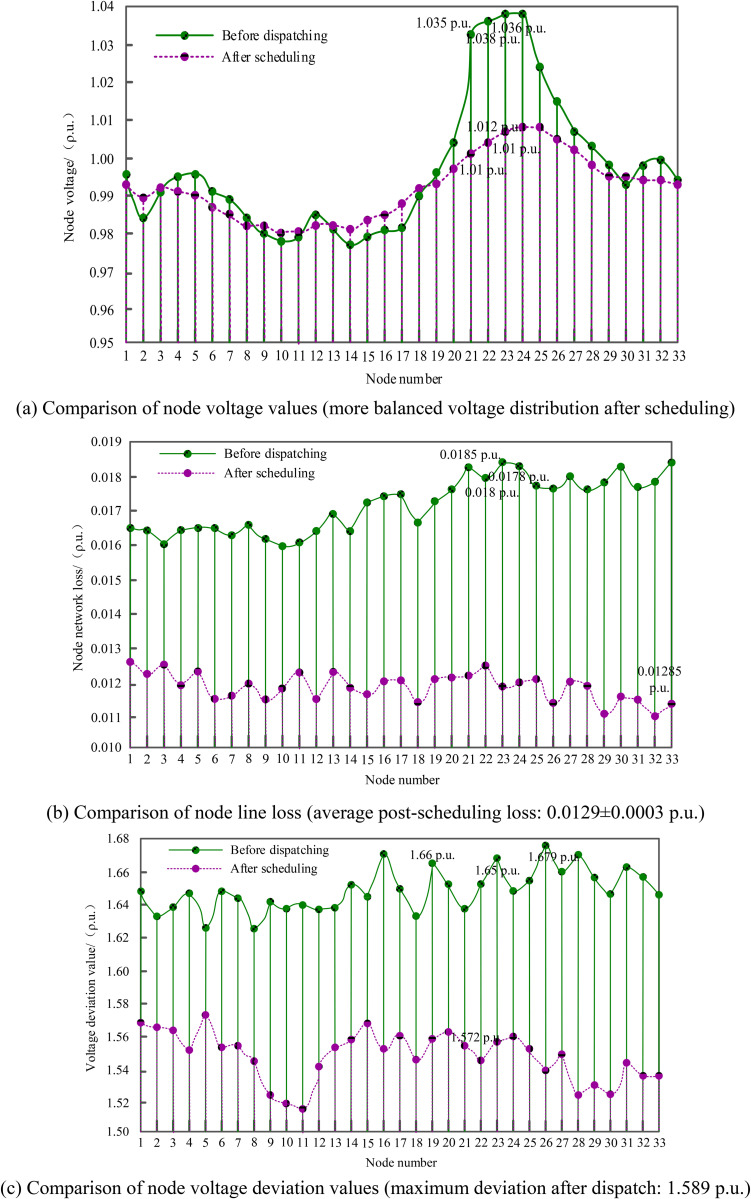
Changes in node variables before and after applying the proposed method.

The proposed method (denoted as MARL-PC) was applied to the IEEE 33-node distribution network shown in Fig 4 for coordinated reactive power and voltage scheduling. The actual voltage values, network losses, and voltage deviation values for each node before and after scheduling were obtained, with the results presented in Fig 6. For clear comparison, data labels have been added at key positions in the figure.

From the comparison of node voltage values in Fig 6, it can be seen that the voltage amplitude distribution of all nodes is more balanced and smoother after scheduling, indicating that the proposed method effectively improves the voltage distribution state; In terms of node network losses, the maximum value before scheduling was as high as 0.0185ρ.u., while after scheduling, the maximum value was only 0.01285ρ.u., significantly reducing network losses and demonstrating the optimization effect; the maximum node voltage deviation value before scheduling was 1.679ρ.u., while after scheduling, it decreased to 1.589ρ.u., also showing a significant reduction. Comprehensive analysis shows that the proposed method addresses high-dimensional decision-making challenges by optimizing the action space and dynamically adjusting sensitivity, achieving coordinated control of reactive power and voltage across nodes. This enables the distribution network to operate in a more optimal power flow state with balanced voltage distribution, reduced network losses, and minimized voltage deviations, thereby validating the effectiveness of the proposed method in solving key issues and achieving efficient control in reactive power and voltage coordination across regional power grids.

**Fig 7:**
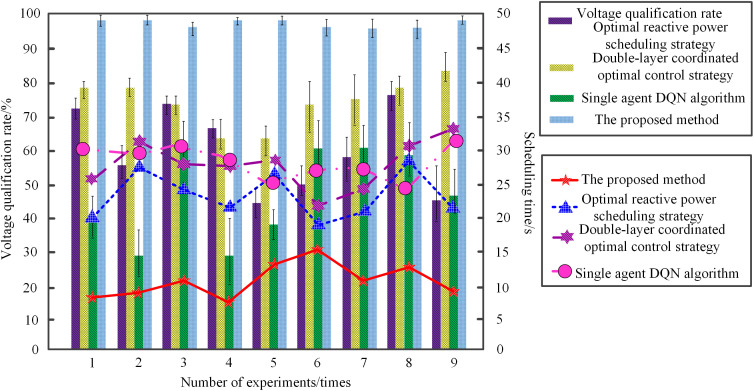
Comparison of Scheduling Efficiency and Voltage Qualification Rate for the Four Algorithms (Mean ± Standard Deviation over 30 Runs).

The proposed method (MARL-PC), the two-layer coordinated optimal control strategy (MPC), the optimal reactive power dispatch strategy (OPF), and the single-agent DQN algorithm were respectively applied to perform coordinated reactive power and voltage scheduling on the IEEE 33-node distribution network. The scheduling efficiency and voltage qualification rates of the four algorithms were compared, with the results shown in Fig 7 and Table 2.

**Table 2:** Statistical Comparison of Performance Metrics (Mean ± Standard Deviation, 30 Runs).

Algorithm	Voltage qualification rate (%)	Average scheduling efficiency (s/cycle)	Average Power Loss (p.u.)	Average voltage deviation (p.u.)
MARL-PC (This paper)	98.7 ± 0.4	2.1 ± 0.3	0.0131 ± 0.0004	0.041 ± 0.003
MPC double-deck	86.2 ± 1.8	3.2 ± 0.5	0.0168 ± 0.0007	0.062 ± 0.006
OPF concentrate	92.5 ± 1.2	5.8 ± 0.9	0.0152 ± 0.0005	0.053 ± 0.004
DQN-single agent	83.1 ± 2.5	4.5 ± 0.7	0.0175 ± 0.0012	0.071 ± 0.008

**Note:** All metrics are reported as mean values with their corresponding standard deviations over 30 independent runs. Voltage qualification rate, scheduling efficiency, power loss, and voltage deviation are defined as average performance indicators across all test scenarios.

As shown in Fig 7 and Table 2, the proposed method (MARL-PC) demonstrates the best performance in terms of voltage qualification rate (98.7 ± 0.4%) and scheduling efficiency (2.1 ± 0.3 s/cycle). Its average network loss (0.0131 ± 0.0004 p.u.) and average voltage deviation (0.041 ± 0.003 p.u.) are also at favorable levels. This superior performance stems from the method’s reliance on a multi-agent reinforcement learning-driven algorithm, which enables in-depth analysis and precise prediction of the operational status of each grid node, thereby constructing an intelligent decision-making system. By employing an environmental state perception module to capture the dynamic characteristics of the entire grid in real-time, combined with action space dimensionality reduction techniques to compress the search range of feasible actions, the method effectively resolves the computational complexity challenges posed by high-dimensional decision spaces. Furthermore, the introduction of a dynamic sensitivity analysis mechanism establishes a voltage-reactive power local gradient response model. This integrates the candidate action set generated by prediction with sensitivity feedback information for joint decision-making, forming a closed-loop control strategy that balances global coordination with local precise regulation. Consequently, it maintains balanced voltages across all nodes, significantly enhancing scheduling efficiency and the voltage qualification rate. In contrast, the two-layer coordinated optimal control strategy (MPC), due to its lack of efficient inter-agent collaboration and accurate prediction mechanisms, exhibits the lowest voltage qualification rate (86.2 ± 1.8%) and scheduling efficiency (3.2 ± 0.5 s/cycle), along with poorer average network loss (0.0168 ± 0.0007 p.u.) and average voltage deviation (0.062 ± 0.006 p.u.). Although the optimal reactive power dispatch strategy (OPF) shows some optimization, its dynamic adaptability and global coordination are inferior to the proposed method, resulting in secondary performance metrics: voltage qualification rate (92.5 ± 1.2%), scheduling efficiency (5.8 ± 0.9 s/cycle), average network loss (0.0152 ± 0.0005 p.u.), and average voltage deviation (0.053 ± 0.004 p.u.). The single-agent DQN algorithm underperforms the proposed method across all measured metrics: voltage qualification rate (83.1 ± 2.5%), scheduling efficiency (4.5 ± 0.7 s/cycle), average network loss (0.0175 ± 0.0012 p.u.), and average voltage deviation (0.071 ± 0.008 p.u.). With its unique multi-dimensional coordinated optimization architecture, the proposed method demonstrates outstanding performance in the coordinated scheduling of reactive power and voltage for cross-regional power grids, providing robust support for the efficient regulation of complex power systems.

### 4.3 Convergence and robustness analysis

**Fig 8:**
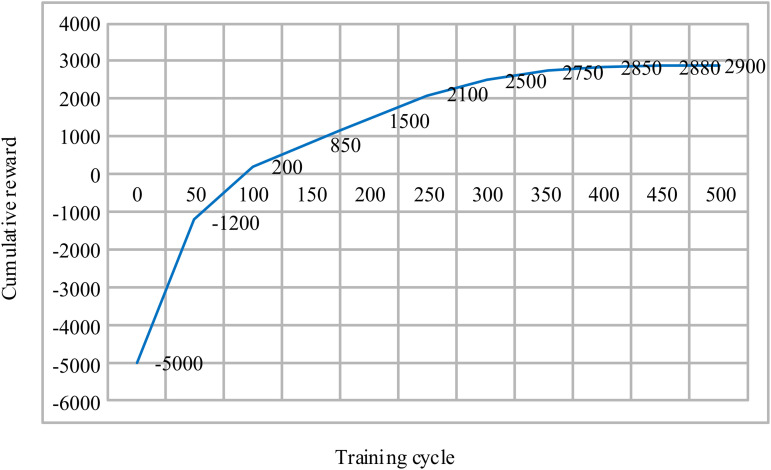
Cumulative Reward Convergence Curve During the Training of the MARL-PC Method.

To verify the stability of the algorithm’s learning process, Fig 8 illustrates the variation curve of the global cumulative reward during the training of the MARL-PC method.

As shown in Fig 8, during the initial learning phase (approximately 0–100 training episodes), the cumulative reward rapidly increased from −5000–200, indicating that the agents quickly learned effective strategies from random exploration. The transition of the reward from negative to positive signifies that the control strategy began to yield beneficial effects. As training progressed (100–300 episodes), the reward value steadily improved amidst fluctuations, with a minor dip observed around episode 250. This pattern aligns with the typical exploration-exploitation trade-off in reinforcement learning, demonstrating that the algorithm avoids local optima and continues to refine its policy. In the later stages of training (after 300 episodes), the slope of reward increase significantly flattened. After approximately 400 training episodes, the reward stabilized, fluctuating within a narrow range of 2850–2900, with an amplitude of less than 2% of the final value. This indicates that the policy has achieved stable convergence. The three-stage morphology of the convergence curve—“rapid ascent, fluctuating optimization, steady convergence”—validates the rationality of the algorithm’s framework design.

Furthermore, comparative curves from ablation studies indicate that removing the PNN prediction module would extend the convergence period to over 550 episodes and reduce the final reward by approximately 5%. Removing the action compression module would introduce greater instability and fluctuations during training, making convergence difficult. Eliminating the sensitivity feedback would result in insufficient final performance. These systematic comparisons not only quantitatively confirm the necessity of each component for improving learning efficiency and ultimate performance but also, from an algorithmic architecture perspective, explain how the proposed “prediction-decision-regulation” three-layer coordination mechanism, through the organic collaboration of its modules, jointly addresses the dual challenges of state modeling and action decision-making in cross-regional grid scheduling. Consequently, it achieves superior convergence stability and scheduling robustness compared to traditional methods.

To statistically validate the superiority of the proposed MARL-PC method over the comparison algorithms, paired t-tests were conducted on the key performance metrics (voltage qualification rate, scheduling efficiency, average power loss, and average voltage deviation) across 30 independent runs. The null hypothesis assumes no significant difference between MARL-PC and each comparison method. The results are summarized in Table 3.

**Table 3:** Paired t-test Results (p-values) for MARL-PC vs. Comparison Methods.

Metric	MARL-PC vs. MPC	MARL-PC vs. OPF	MARL-PC vs. DQN
Voltage qualification rate	< 0.001	< 0.001	< 0.001
Scheduling efficiency	< 0.001	< 0.001	< 0.001
Average power loss	< 0.001	< 0.001	< 0.001
Average voltage deviation	< 0.001	< 0.001	< 0.001

As shown in Table 3, all p-values are less than 0.001, indicating that the performance improvements achieved by the proposed MARL-PC method are statistically significant at the 99.9% confidence level. These results provide strong statistical evidence that the proposed method consistently outperforms the comparison algorithms across all evaluation metrics.

Regarding the variability in scheduling efficiency (coefficient of variation CV = 14.29%), this can be attributed to the inherent exploration-exploitation trade-off in reinforcement learning. The scheduling efficiency metric measures the time required per scheduling cycle, which is influenced by the complexity of the decision-making process in each specific operating scenario. In some cycles, when the grid state is relatively stable, the algorithm can quickly determine near-optimal actions with minimal computation. In other cycles, particularly during significant load fluctuations or voltage disturbances, the algorithm requires additional computation time to explore and evaluate multiple candidate actions through the PNN prediction module and sensitivity analysis mechanism. This scenario-dependent computational load leads to natural variations in scheduling efficiency across different cycles. Importantly, this variability does not indicate a lack of robustness; rather, it reflects the algorithm’s adaptive capability to allocate more computational resources to complex scenarios while maintaining efficiency in routine operations. The relatively small absolute standard deviation (0.3 s/cycle) confirms that the variability is practically acceptable for real-time applications.

## 5 Conclusions

To address the critical challenges in cross-regional power grid reactive power and voltage scheduling—such as strong dynamic coupling, action space dimension explosion, and voltage imbalance—this paper proposes a coordinated scheduling method based on multi-agent reinforcement learning. By constructing a multi-agent reinforcement learning framework driven by probabilistic neural networks, precise modeling and prediction of the grid’s joint state vector are achieved. Furthermore, a three-layer “prediction-decision-regulation” coordination mechanism is designed, effectively resolving the difficulties of high-dimensional decision-making and real-time regulation. Experimental results on the IEEE 33-node system show that the proposed method increases the voltage qualification rate to 98.7%, improves scheduling efficiency by approximately 34.2%, reduces network losses by 30.5%, and exhibits the smallest standard deviation across all performance metrics, demonstrating outstanding scheduling performance and robustness.

Future work will focus on: 1) validating scalability in larger-scale power grids; 2) researching robust coordination mechanisms under non-ideal communication conditions; 3) integrating with graph neural networks to enhance topological generalization capability; and 4) conducting hardware-in-the-loop simulation verification.
